# Association of Graphene Silver Polymethyl Methacrylate (PMMA) with Photodynamic Therapy for Inactivation of Halitosis Responsible Bacteria in Denture Wearers

**DOI:** 10.3390/nano11071643

**Published:** 2021-06-23

**Authors:** Cecilia Bacali, Rahela Carpa, Smaranda Buduru, Mirela L. Moldovan, Ioana Baldea, Annemarie Constantin, Marioara Moldovan, Doina Prodan, Laura Monica Dascalu (Rusu), Ondine Lucaciu, Florinela Catoi, Mariana Constantiniuc, Mandra Badea

**Affiliations:** 1 Department of Prosthodontics and Dental Materials, Iuliu Hatieganu University of Medicine and Pharmacy, Clinicilor 32, 400006 Cluj-Napoca, Romania; cecilia.bacali@yahoo.com (C.B.); smarandabuduru@yahoo.com (S.B.); rusu.monica.laura@gmail.com (L.M.D.); mconstantiniuc@umfcluj.ro (M.C.); 2 Department of Molecular Biology and Biotechnology, Faculty of Biology and Geology, Babeș Bolyai University, 1 M. Kogălniceanu Street, 400084 Cluj-Napoca, Romania; 3 Department of Dermopharmacy and Cosmetics, Faculty of Pharmacy, Iuliu Hatieganu University of Medicine and Pharmacy, 12 I. Creanga Street, 400010 Cluj-Napoca, Romania; mmoldovan@umfcluj.ro; 4 Department of Physiology, Iuliu Hatieganu University of Medicine and Pharmacy, Clinicilor, 400006 Cluj-Napoca, Romania; baldeaioana@gmail.com; 5 Department of Morphological Sciences, Iuliu Hatieganu University of Medicine and Pharmacy, 6 L. Pasteur, 400335 Cluj-Napoca, Romania; annemarie_chindris@yahoo.com; 6 Raluca Ripan Institute for Research in Chemistry, Fantanele 30, Babeș Bolyai University, 400294 Cluj-Napoca, Romania; mmarioara2004@yahoo.com (M.M.); doina_prodan@yahoo.com (D.P.); 7 Department of Oral Health, Iuliu Hatieganu University of Medicine and Pharmacy, Avram Iancu 31, 400083 Cluj-Napoca, Romania; ondineluc@yahoo.com; 8 Department of Functional Biosciences, Iuliu Hatieganu University of Medicine and Pharmacy, Avram Iancu 31, 400083 Cluj-Napoca, Romania; adriana.catoi@umfcluj.ro; 9 Department of Preventive Dental Medicine, Iuliu Hatieganu University of Medicine and Pharmacy, Avram Iancu 31, 400083 Cluj-Napoca, Romania; mebadea@umfcluj.ro

**Keywords:** halitosis, graphene, photodynamic therapy, *Enterococcus faecalis*, *Porphyromonas gingivalis*

## Abstract

(1) Background: Poor hygiene and denture presence in the oral cavity are factors that favor bacterial accumulation, the cause of halitosis and of various oral and general diseases. Aim: This study aimed to evaluate the possibility of inactivating bacteria associated with halitosis in acrylic denture wearers using polymethyl methacrylate resin enhanced with graphene silver nanoparticles and the effect of the resin association with extra oral photodynamic therapy. (2) Methods: Graphene silver nanoparticles in 1 and 2 wt% were added to a commercial acrylic resin powder. Three study groups containing samples from the three different materials were established. The first group was not exposed to the light treatment, and the other two were exposed to red light (laser and light emitting diode) after photosensitizer placement on the disk’s surface. Samples were incubated with *Porphyromonas gingivalis* and *Enterococcus faecalis*. (3) Results: For both bacterial strains, inhibition zones were obtained, showing significant differences for the light-treated samples. (4) Conclusions: Denture resins with antibacterial properties associated with extra oral photodynamic therapy exhibited enhanced antibacterial effects. The procedure could be used as a safer and more efficient alternative technique against halitosis and oral infections in denture wearers.

## 1. Introduction

Inadequate oral hygiene, characterized by accumulation of food debris, increased denture plaque, and tongue deposits in denture wearers is the major cause for oral malodor by promoting bacterial accumulation in the oral cavity. Denture plaque is a reservoir of pathogenic bacteria that can be associated with different oral conditions and with major geriatric health problems [1,2]. Microbial intensive growth is favored by the tongue and saliva-reduced cleaning actions and by the anaerobic habitat found under the denture base. 

Significant associations between bad breath and tongue deposits, denture quality, overnight denture wearing, systemic diseases, and medicine intake were found [3]. The prevalence of volatile sulphur compounds like hydrogen sulphide (H_2_S) was found to be significantly higher among denture wearers compared with subjects without dentures, with H_2_S being associated with tongue deposits and overnight denture wearing, while dimethyl sulphide (CH_3_)_2_S was associated with systemic diseases and the intake of medicines [3]. An extreme reduction of resting salivary flow and increased tongue coating were also associated with higher levels of H_2_S and methyl mercaptan (CH_3_SH) [4]. Decreased salivary flow in the case of systemic conditions, medication, or during nighttime can increase the intensity of halitosis [5]. 

Gram-negative oral bacteria are considered the main factor responsible for the production of volatile sulphur compounds associated with oral malodor—methyl mercaptan, hydrogen sulphide, dimethyl sulphide—and other malodorous substances (diamines as putrescine and cadaverine or butyric, valeric, or propionic acids) [6,7]. *Enterococcus faecalis* proved to be one of the predominant bacteria present in removable-denture wearers and was also associated with halitosis [8]. Higher amounts of Enterobacteriaceae are present in the oral environment of denture wearers (48%), compared with the normal population (16.4%), and the incubation of this bacterial strain on a sterile denture was shown to produce the characteristic denture odor. *Porphyromonas gingivalis* is another main bacterium involved in oral malodor [9].

Although an improvement of oral hygiene can be obtained by mechanical methods such as brushing or by chemical methods that use mouthwash solutions or cleansing tablets with antimicrobial effects, they have limited efficiency [10]. 

Polymethyl methacrylate (PMMA) is the most often-used material in denture fabrication, but it has several shortcomings, among them being the strength [11] and the antibacterial activity. Studies showed that bacterial adhesion to polymethyl methacrylate surfaces seems to be reduced when compared with other acrylic denture base materials, like polyethylene terephthalate (PET), and consequently PMMA would be more recommended for patients with inadequate hygiene and halitosis [12]. However, the presence of the acrylic materials in the oral cavity enables bacterial adhesion and biofilm formation, also favoring inflammation of oral tissues and halitosis [13]. 

The finishing and polishing procedures can influence the roughness and gloss of resin-based materials and can also have an important role in biofilm formation [14]. Studies showed that material composition and characteristics play a bigger role in bacterial adhesion than the surface roughness [15]. Addition of different nanoparticles can improve the acrylic resins’ properties (antibacterial, antifungal, and mechanical properties), as was shown in many studies [16,17,18].

In the recent years, the effect of nanofiller addition to different materials was evaluated because of an increasing interest towards medical [19,20] and dental materials [21] and antimicrobial and mechanical properties improvement. The addition of silver nanoparticles to acrylic resins has shown an efficient antimicrobial action [22], while the addition of silver nanoparticles and graphene improved both mechanical [18] and antimicrobial properties of a denture resin [23].

Photodynamic therapy (PDT) is a minimally invasive and minimally toxic treatment for destroying bacteria or diseased cells, and is based on a photochemical reaction that uses a light sensitive photosensitizer. The photo-sensitive substance is activated by exposure to a specific light beam before toxic products for the targeted cells are released [24]. The antibacterial effect is possible due to the production of reactive oxygen species, especially singlet oxygen [24]. PDT destroys selective cells in a short time, can be applied to wide surfaces, and can be repeated easily, with portable devices being available [25]. Visible light was also shown to have effects on the odoriferous components [26]. In a study which assessed the effect of visible light against *Porphyromonas gingivalis*, a reduction in the bacterial count was obtained, which suggested the possibility of using PDT as a prophylactic treatment with no side effects [27]. However, photosensitizing effects of PDT on the skin tissues were reported [28]. Other early or late onset side effects, such as pain, burns, erythema, and desquamation can occur following PDT, causing discomfort for the patient [25]. 

The possibility of inactivating the halitosis-responsible bacteria using improved denture materials and also the new approaches of enhancement of the acrylic resin’s antibacterial effect using associations of safe techniques could be highly beneficial for denture or orthodontic device-wearing patients. The aim of this study was to evaluate the possibility of inactivating oral bacteria associated with halitosis in acrylic denture wearers using polymethyl methacrylate (PMMA) resin enhanced with graphene silver nanoparticles with and without the association of extra oral photodynamic therapy (PDT). The null hypothesis was that the enhanced resins have no effect on halitosis-responsible bacteria. 

## 2. Materials and Methods

### 2.1. Acrylic Samples Preparation

Graphene silver nanoparticles were synthetized by the radio frequency catalytic chemical vapor deposition (RF-CCVD) method, using silver nanoparticles distributed over magnesium oxide (Agx/MgO, where x = 3 wt%) as described in a previous study [29]. Then, they were added to a commercial acrylic resin powder, Castavaria (Vertex Dental, Zeist, The Netherlands), in 1% and 2% weight percentages, using a lab-vibrating device [23]. After mixing the monomer and the polymer in the recommended ratio (1 mL/0.95 g monomer: 1.7 g polymer), the material was poured in a silicone mold (stick shaped) and cured for 30 min at 55 °C and 2.5 Barr in a polymerizing unit, according to the producer’s specifications, resulting sticks of 15 × 5 mm. Disks 5 mm in diameter and 1mm in thickness were obtained by cutting the sticks with a cutting device (Isomet 1000, Precision Saw, Buehler, ITW, IL, USA) and divided into three study groups: C (control—simple resin), P1 (resin with 1 wt% graphene silver nanoparticles), and P2 (resin with 2 wt% graphene silver nanoparticles).

### 2.2. Acrylic Samples Investigations

#### 2.2.1. Cross Polarized Light Microscopy (CPOM)

The powders used in the study were characterized by Cross Polarized Light Microscopy (CPOM) to observe in which manner graphene-silver powder coated the polymer spheres. Therefore, the investigation was performed on a Laboval II Mineralogical Microscope (Karl Zeiss Jena, Oberkochen, Germany) equipped with a Samsung digital camera 8 MPx, (Samsung, Seoul, Korea).

#### 2.2.2. FT-IR Spectroscopy

FT-IR Spectroscopy was used to detect the functional groups, specific for the studied materials. An Able Jasco 610 instrument (ABL&E-JASCO Group, Wien, Austria) in the range of 4000 to 500 cm^−1^ was used.

#### 2.2.3. Scanning Electron Microscopy

The investigation was performed at 10,000× magnitude in a low-vacuum, pressure of 80 Pa, at 15 kV and 11 mm working distance.

### 2.3. In Vitro Testing of the Acrylic Samples’ Toxicity

*Cell cultures.* The assays were performed on human gingival fibroblasts—HGF (ATCC CRL-2014, HGF-1, LOT: 63449675) purchased from ATCC (Manassas, VA, USA). The cell culture medium was DMEM, supplemented with 10% FBS (Fetal Bovine Serum), antibiotics, and antimycotics. All reagents were purchased from Sigma Aldrich, Co., Heidelberg, Germany.

*Extract preparation*. The samples from all three studied groups were sterilized by immersion in ethanol 70° for 30 s and then washed three times in sterile PBS. The disks were then incubated for 24 h at 37 °C with the cell culture medium at a concentration of 3 cm^2^/mL, complying with the ISO 10993-12/2012 procedures [30]. The extract was immediately used for the experiments. 

*Viability assay*. Cell survival was assessed using CellTiter 96^®^ AQueous Non-Radioactive Cell Proliferation Assay (Promega Corporation, Madison, WI, USA). The cells were cultivated at a density of 104/well in 96-well plates (TPP, Trasadingen, Switzerland) for 24 h, then exposed to extracts of the samples diluted in medium for 24 h at a dilution range of 0–64×. Viability was measured by colorimetric analysis using an ELISA plate reader (Tecan, Männedorf, Switzerland) at 540 nm. All experiments were done in triplicate. Untreated cell cultures were used as controls. Results are presented as OD540.

*Phaloidin staining*. Phalloidin—FITC 50 µg/mL (Sigma Chemical Co., St. Louis, MO, USA) a marker for actin myofilaments (green)—was used according to the manufacturer’s instructions. HGF cells were seeded in chamber slides at a density of 5 × 10^4^/chamber, allowed to settle for 24 h, and then exposed to the undiluted extract of each sample. Controls were exposed only to the medium. The treated cells were then stained with phalloidin-FITC. Images of cells were documented using an inverted microscope Olympus BX40 equipped with an Olympus CKX-RFA fluorescent lamp and an E330 camera (Olympus, Hamburg, Germany) at a magnification of 20×. 

### 2.4. Antibacterial Assays

The microorganisms tested in this study were *Porphyromonas gingivalis* ATCC 33277 and *Enterococcus faecalis* ATCC-29212, from the Microbiology Lab, Faculty of Biology and Geology, UBB, Cluj-Napoca. Diffusometric analysis was used for the evaluation of the antimicrobial activity. Aseptic disks 5 mm in diameter and 1 mm in thickness prepared from the three types of materials were used as acrylic resin samples. Bacterial suspensions from the fresh strains were prepared in normal saline solution and adjusted to a 0.5 McFarland turbidity. Mueller-Hinton culture medium (Oxoid, UK) was used to inoculate the bacterial suspensions [31]. 

After the samples were made, they were divided into three study groups, each of them containing C (simple resin), P1 (resin with 1% graphene silver nanoparticles), and P2 (resin with 2% graphene silver nanoparticles). The samples obtained from the resin without the addition of nanoparticles were used as control. The first study group was not exposed to light treatment; the second group was exposed to diode light after FotoSan Agent placement on the disk surface and the third group was exposed to laser light after the placement Helbo Blue Photosensitizer on the sample surface. The disks were exposed to the light for 3 min. A portable light emitting diode FotoSan 630 (CMS Dental, 630 nm wavelength, 2000–4000 nw/cm^2^) and a therapeutic red laser, Helbo TheraLite Laser (Bredent Medical GmbH&CO, Germany, 660 nm wavelength), were used as light sources.

The disks from each group were then placed on plates seeded separately with the two bacterial strains to assess their antimicrobial activity. After incubation at 37 °C for 48 h, the inhibition zones were measured using an inhibition-zone-measuring scale. All assays were performed in triplicate, under aseptic conditions.

### 2.5. Scanning Electron Microscopy (SEM) Investigations of the Bacterial Cells

*Porphyromonas gingivalis* and *Enterococcus faecalis* incubated for 24 h at 37 °C were analyzed using scanning-electron microscopy. After the incubation, preparations were completed and then fixed on the layer of the double-adhesive carbon strip on the mount in the Chemistry Lab of Raluca Ripan Institute for Research in Chemistry. The examination of the surface microstructure was performed using an Inspect S Scanning Electron Microscope (FEI Company, Eindhoven, The Netherlands). The determinations were performed in a low vacuum, at a pressure of 80 Pa, at 15 kV and a work distance of 11 mm. 

### 2.6. Statistical Analysis

The statistical differences between the experimental groups were evaluated by one-way ANOVA and the paired Student t test, followed by the Bonferroni posttest using GraphPad. Results were considered significant for *p* ≤ 0.05. The statistical package used for data analysis was Prism version 4.00 for Windows, GraphPad Software, San Diego, CA, USA. 

The statistical difference between the antimicrobial activity of the samples (*n* = 5) were evaluated using one-way ANOVA and the Tukey test for post hoc comparisons between groups. The significance level was set at *p* = 0.05. Origin2019b Graphing and Analysis software was used. All the values in text and figures are expressed as mean ± standard deviation. The results were considered significant for *p* ≤ 0.05.

## 3. Results

### 3.1. Acrylic Samples Investigations 

#### 3.1.1. Cross Polarized Light Microscopy

**Figure 1:**
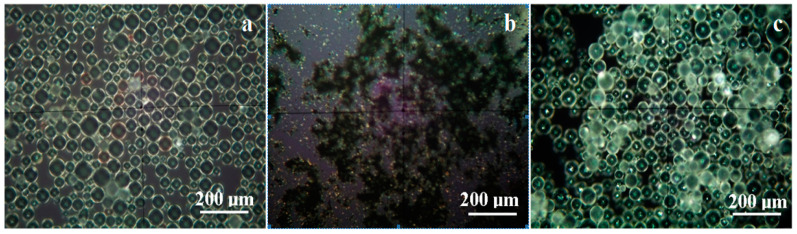
Cross-polarized light microscopy images of the powders: (**a**) polymer spheres (M), (**b**) graphene-silver, and (**c**) polymer spheres coated with graphene-silver (P2).

The biologic effect of the experimental materials was in a close connection with the micro-structural disposal of the graphene-silver powder on the polymer spheres. CPOM images in Figure 1 provide a proper characterization of the coating process. 

Polymer powder consists of micro-sized spheres with diameters in a range of about 20 to 50 µm, Figure 1a. Their aspect features a bright white of the circumference border and a pale-shadow halo inside. The aspect is correlated with the low crystallinity of the polymer.

Graphene-silver powder has a complex mixture of very fine graphene particles organized in micro-sized clusters with a dendrite shape and sizes ranging from 5 to over 100 µm, Figure 1b. Their aspect is black, related to a very low crystallinity due to the nano structural organization within the graphene grains. Silver particles appeared as bright yellow dots with diameters predominantly between 1 to 2.5 µm, but significant submicron dots were observed, Figure 1b. The bright yellow aspect of silver micro and nano particles observed by CPOM was caused by the high crystallinity within them, a fact also reported in the literature [32,33]. 

Mixing the polymer with graphene-silver powder facilitates the smallest-particles adsorption on the sphere’s surface. Figure 1c shows many spheres with a bright and compact black aspect due to the strong and uniform adhesion of the grapheme on the polymer surface. Silver particles having smaller diameters compared with the spheres is also attached to their surface being observed as bright spots. Some of the polymer spheres appear to remain uncoated, having the same halo aspect as observed in Figure 1a.

#### 3.1.2. FTIR Spectroscopy

The FTIR spectrum of PMMA had a peak at 1732 cm^−1^ that was due to acrylate carboxyl groups; at 1388 cm^−1^, this was due to O–CH_3_ bending vibrations, and the peak at 1242 cm^−1^ was assigned to the twisting mode of the –CH_2_ group in PMMA. The FTIR peak of PMMA at 1439 cm^−1^ was assigned to the bending of C–H bonds in the –CH_3_ group [34]. 

**Figure 2:**
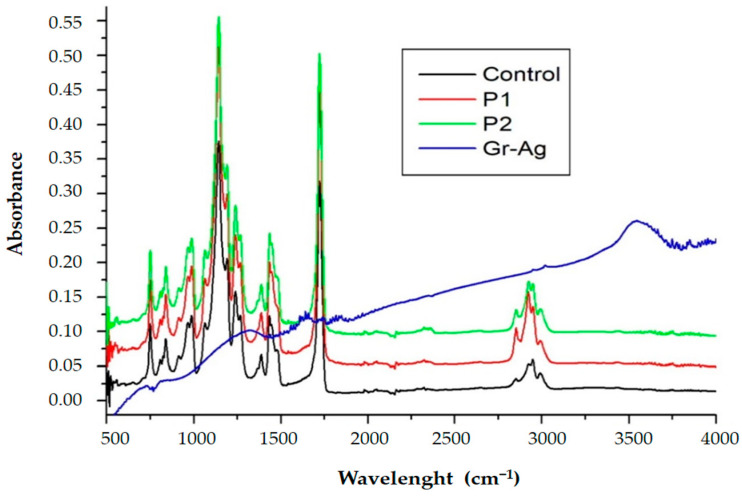
FTIR spectra of the PMMA polymer (enhanced with G-Ag and control).

The infrared absorption spectrum of G-Ag was characterized by the existence of stripes with different peaks. The highest peak was at 3430 cm^−1^ (stretching and bending vibrations of –OH group), and 1275 cm^−1^ was assigned to C–OH, while the peak centered at 1600 cm^−1^ was assigned to C=C bonds associated to the skeletal vibrations of unoxidized graphitic domains. The peak at 1720 cm^−1^ was assigned to C=O bonds in the fragments of carboxylic acid and carbonyl. The G-Ag nanoparticles exhibited a new stripe at 1420 cm^−1^, resulting from the reduced C–N stretching vibration of G-Ag. The peak at 1.320 cm^−1^ was assigned to C–O–C. Further, the peak of absorbance at about 1.270 cm^−1^ was assigned to the C–OH bond, and the new absorbance stripe at 1570 cm^−1^ was assigned to the skeletal vibration of the graphene sheets. The spectra of P1 and P2 samples showed the presence of the stripes specific to both PMMA and G-Ag (Figure 2).

#### 3.1.3. Scanning Electron Microscopy Investigations of the Acrylic Samples

**Figure 3:**
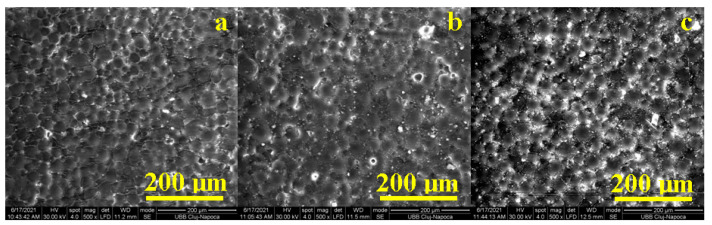
SEM images of the acrylic samples: (**a**)—PMMA, (**b**)—PMMA + 1%GO and (**c**)—PMMA + 2%GO.

The dispersion and alignment of graphene nanoparticles into a polymer matrix play a key role in the properties of materials. In this regard, we characterized the PMMA-Graphene by using scanning electron microscopy (SEM). The SEM images of the nanocomposite are shown in Figure 3a–c to certify the even dispersion of the graphene within the PMMA matrix. According to these images, nanoparticles were well dispersed in the polymer phase as a function of the added percent (Figure 3b. 1% and Figure 3c 2%). SEM images showed that PMMA-graphene composites had surface morphology similar to PMMA (Figure 3a) composites, as shown in Figure 3b,c.

### 3.2. Cell Viability

**Figure 4:**
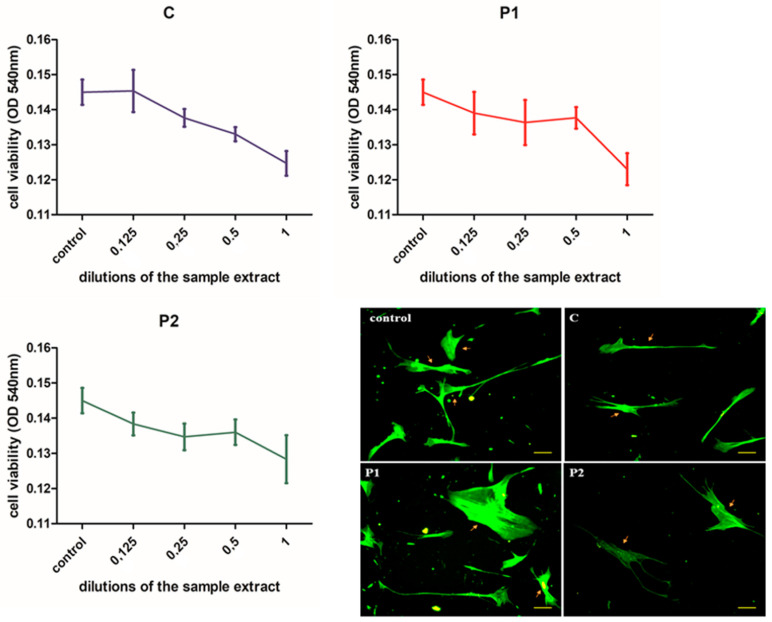
Cell-viability testing in the study groups based on dilutions of the sample extract. Cells were exposed to binary dilutions of the sample extract (1/8, 1/4, ½ and undiluted) for 24 h and viability was evaluated by colorimetrical measurement of formazan production. Results are presented as OD. C = control sample, P1 = resin with 1 wt% graphene silver nanoparticles, P2 = resin with 2 wt% graphene silver nanoparticles. Comparative images of HGF cells treated with undiluted extract of each sample and then stained with Phaloidin-FITC are presented, bar = 20 µm. Yellow arrows show the normal morphological aspect of the HGF cells in all the groups.

As seen in Figure 4, the cells’ viability was slightly decreased when exposed to the undiluted sample extract, though the decrease was not significant. Additionally, there were no significant viability differences between the experimental groups, as shown by one-way ANOVA (*p* = 0.98) and the Bonferroni posttest (*p* = 0.191, C vs. P1, *p* = 0.137, C vs. P2 and *p* = 0.054, P1 vs. P2). The decrease was above 80% of the control values for samples C and P1 and above 90% for sample P2. These results showed that the materials were rather well tolerated by the cells with no toxicity (viability below 70% of untreated control was considered toxic level).

Phaloidin staining showed no alteration of the cytoskeleton as compared to the untreated control in all the treated groups. In all groups, the cells had a normal morphological aspect, they were adherent to the substrate, and they had multiple cytoplasmatic elongations, without signs of cellular distress. 

### 3.3. Antimicrobial Effect of the Acrylic Samples

**Figure 5:**
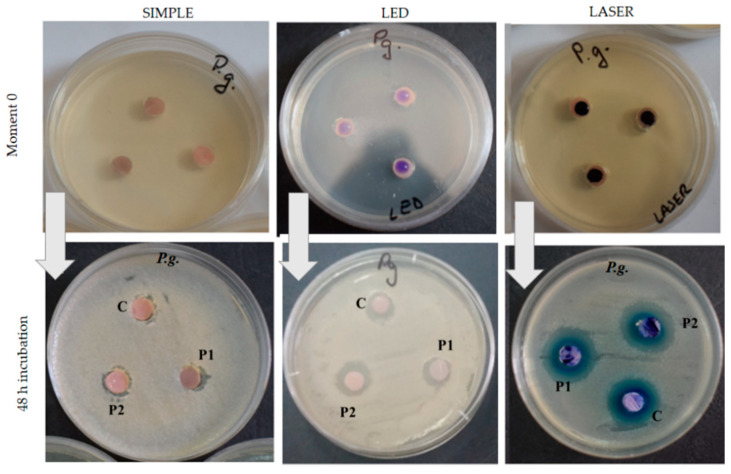
Antimicrobial activity of the samples (placed on the culture medium) against *Porphyromonas gingivalis* (Pg) in 48 incubation hours. (Three experimental variants: simple variant = without light treatment; LED-treated variant; Laser-treated variant).

**Figure 6:**
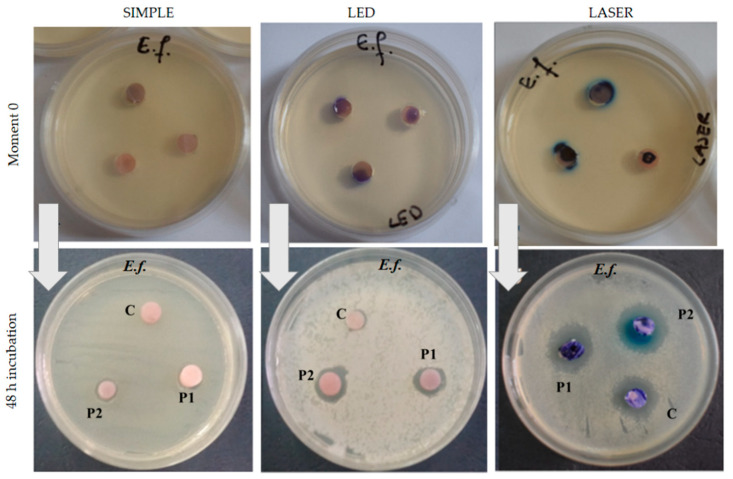
Antimicrobial activity of the samples (placed on the culture medium) against *Enterococcus faecalis* (Ef) in 48 incubation hours. (Three experimental variants: simple variant = without light treatment; LED-treated variant; Laser-treated variant).

Antimicrobial activity was tested on three experimental groups: simple variant without light treatment, LED-treated variant, and Laser-treated variant. After incubation, for both bacterial strains, *Porphyromonas gingivalis* (Figure 5) and *Enterococcus faecalis* (Figure 6) large-inhibition zones were obtained. Significant differences were observed between the light-treated resins and the other specimens.

The larger inhibition zones were found in P1 and P2 samples (PMMA enhanced with 1 or 2% nanoparticles) when the photosensitizer was placed on their surfaces and the resins were exposed to laser light (for both bacteria). Higher inhibitory effect was obtained for *Porphyromonas gingivalis* than for the *Enterococcus faecalis* (Figure 6) strain in all the study groups.

**Figure 7:**
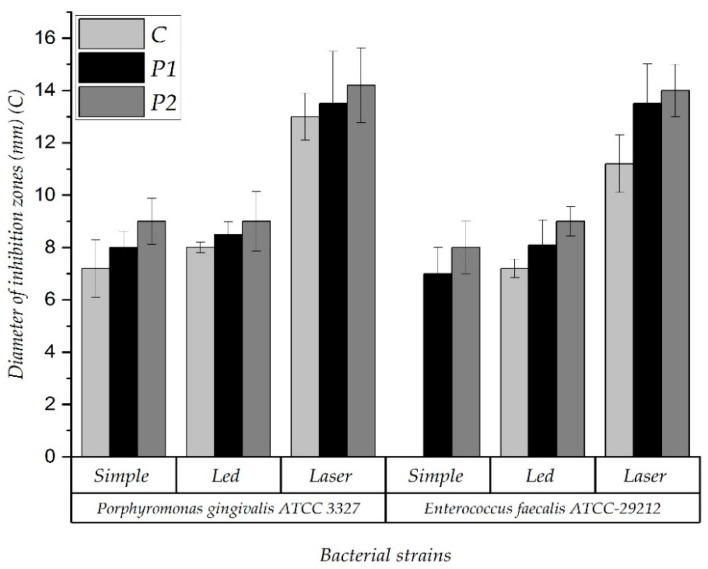
The antimicrobial activity of the samples in different experimental variants (simple, LED, and Laser) for *Porphyromonas gingivalis* ATCC 33277 and *Enterococcus faecalis* ATCC 29212 P1 and P2 samples compared to control (C).

It was noticed that the diameter of the inhibition zone was larger for the P2 sample compared with the other ones. Additionally, the inhibition zone was much larger for *Porphyromonas gingivalis* than for *Enterococcus faecalis*. The laser-light treatment showed the largest inhibition effect in all experimental groups, for both bacterial cultures (Figure 7). 

The antimicrobial activity of the P1 samples, in different experimental variants (simple, LED, and Laser) for *Porphyromonas gingivalis* ATCC 33277 and *Enterococcus faecalis* ATCC 29212, compared to control (simple variant), were not significantly different (*p* = 0.38156). The same results were observed from P2 and C comparison, with *p* = 0.22785, without statistical differences.

### 3.4. Scanning Electron Microscopy Investigations of the Bacterial Cells

**Figure 8:**
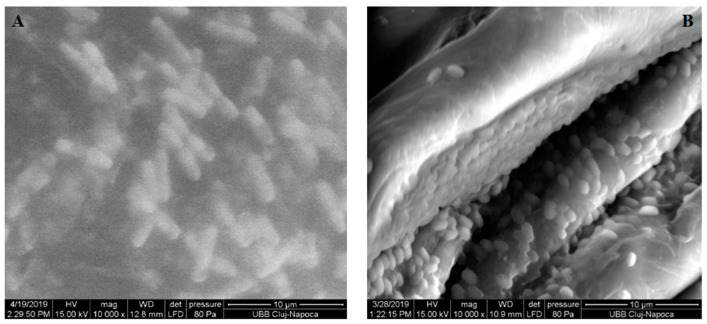
SEM investigation at 10,000× magnitude for (**A**) *Porphyromonas gingivalis* ATCC 33277 and for (**B**) *Enterococcus faecalis* ATCC 29212.

The aspect of the bacterial cells was analyzed on SEM images: rod shaped cells for *P. gingivalis* and ovoid cells for *E. faecalis* (Figure 8).

## 4. Discussion

Like other bacteria that cause oral infections, halitosis-responsible bacteria have evident associations with denture wearers’ discomfort. Still, there are no established protocols for the diagnosis and treatment of bad breath [35]. Halitosis in denture wearers is favored on one hand by poor oral hygiene and on the other hand by the presence of the denture (denture base-material characteristics, and of the saliva and tongue-reduced cleaning action). Systemic diseases together with impaired motility and memory loss can also influence the oral hygiene status in the elderly population [36]. 

In this study, possibilities to inactivate halitosis responsible bacteria using enhanced materials with and without association with photodynamic therapy were evaluated. PMMA acrylic resin enhanced with graphene silver nanoparticles showed antimicrobial effects on the studied bacterial strains, especially when using higher concentrations of nanoparticles (2%). Higher efficiency was found for the association of the studied resins with PDT and in particular the laser light. The studied resins were well tolerated by gingival fibroblasts, and cell viability remained above the toxic level after exposure to the samples.

Several bacterial species, mainly Gram-negative bacteria that colonize denture or dental plaque, are responsible for bad breath and periodontal disease: *Porphyromonas gingivalis, Fusobacterium nucleatum, Prevotella intermedia, Solobacterium moorei, Prevotella (Bacteroides) melaninogenica, Treponema denticola, Porphyromonas endodontalis, Bacteroides loescheii, Enterobacteriaceae, Tannerella forsythensis (Bacteroides forsythus), Centipeda periodontii, Eikenella corrodens, Fusobacterium nucleatum vincentii, Fusobacterium nucleatum, Fusobacterium nucleatum polymorphum, and Fusobacterium periodonticum* [37]. All these bacterial species can decompose proteins to aminoacids as methionine and cysteine in the oral cavity. These aminoacids are further metabolized, resulting in volatile sulphur compounds [38]. 

The bacterial strains used in the study were *Porphyromonas gingivalis* and *Enterococcus faecalis. Porphyromonas gingivalis* is a black-pigmented, Gram-negative, anaerobic, nonmotile, and pathogenic bacteria which belongs to the phylum Bacteroidetes [39]. At SEM investigation, *P. gingivalis* appeared as rod-shaped (Figure 7). *Enterococcus faecalis* is a Gram-positive, catalase-negative, fermentative, non-spore-forming, facultative, anaerobic bacteria. At SEM, their cells were ovoid and about 0.5–1 μm in diameter (Figure 7). They appear alone, in pairs, or in short chains and most strains are nonhemolytic and nonmotile [40]. Denture resins’ surface chemistry and topography can favor bacterial adhesion [41]. Many authors aimed to improve the characteristics of denture resins by adding different materials to obtain antibacterial properties. The addition of graphene and silver nanoparticles to denture-base resins showed favorable effects against different bacterial strains [16], and materials with higher concentrations of additives exhibited better antimicrobial activity against the studied bacteria [23,42]. As both silver and graphene nanoparticles were shown to exhibit antibacterial effects, their association in this study was used in order to obtain an enhanced antimicrobial activity [11,43,44,45]. Graphene was also used for its anchoring and stabilizing effect for the silver nanoparticles. The percentage of graphene silver additives was selected to be 1 and 2%, as the best results for resins reinforcement were reported for low nanofiller contents [46] and because of the color changes in the resins caused by their black color. For 1% addition of fillers, the color changes were discreet, while for the 2% loading the color changes were more evident. Higher concentrations could significantly affect the esthetics in visible areas. For these concentrations of additives, significant improvements of mechanical properties of the acrylic resin were obtained [18,23].

Photodynamic therapy (PDT), or light-activated therapy, is a treatment that uses a powerful light with a specific wavelength in association with a photosensitizer. PDT is a minimal and noninvasive treatment that was shown to significantly restrict the growth of or even to kill the microbes by photochemical reaction [47]. The photosensitive agent absorbs energy from the light and transfers it to oxygen, resulting in highly reactive oxygen species (ROS) that are effective against bacteria, fungi, and viruses [48,49]. Although PDT efficiency was mostly studied in tumor (malign cells) treatment, it has significant potential to be used in many preclinical and clinical fields [50]. PDT showed good results in the treatment of denture stomatitis [51], being more efficient on denture bases than on palatal mucosa [52]. 

Several studies were directed towards the efficiency of PDT against oral bacteria [53]. Other studies assessed the effect of the visible light irradiation without photosensitizers on bacteria, finding that light irradiation had a phototoxic effect on Gram-negative species, such as *Porphyromonas gingivalis* [54,55]. Chan demonstrated that a 60 s exposure to a 100 mW laser light at 665 nm in the presence of methylene blue eliminated up to 99–100% of *Porphyromonas gingivalis* cultures [56]. It has also been proved that PDT helps healing and the cleaning of oral tissues, including the root canals, by eliminating highly resistant species such as *Enterococcus faecalis*. A study that assessed the effect of PDT, using toluidine blue and a 635 nm light source on *Enterococcus faecalis*, showed a significant reduction in bacterial growth, although it was less effective than the combination of sodium hypochlorite (NaOCl) irrigation and PDT in endodontic infections [57]. Lopez-Jimenez et al. demonstrated a reduction in *Enterococcus faecalis* biofilms, with severe damage and cell lysis on AFM images, when using methylene blue and a 670 nm diode laser or toluidine blue with a 628 nm LED light for 30 s [58]. Additionally, laser irradiation was effective in sealing dentinal tubules and eliminating bacteria that had penetrated to the depths of dentin [59]. 

Although PDT is generally considered a safe antibacterial approach without side reactions on oral tissues according to Komerick et al. [60], many studies reported pain and other side effects caused by the procedure [61]. In the present study, we aimed at assessing the effect of the photosensitive agent placed on PMMA (control and enhanced samples) and activated it outside the mouth before the contact with the studied bacterial strains in order to prevent pain and other side effects that can appear during direct-tissue exposure to red light. 

The results obtained in the study showed that PMMA enhanced with graphene silver nanoparticles exhibited a higher inhibitory effect on halitosis responsible bacteria compared with control, with increased antimicrobial activity on the *Porphyromonas gingivalis* strain and when using higher concentrations of additives (2 wt%). In the absence of the light treatment, the control had no effect on the *Enterococcus faecalis* strain, but exhibited a low antimicrobial activity on the *Porphyromonas gingivalis strain*. This could be caused by the polymer degree of conversion, the residual monomer, or the resin cytotoxicity [62]. Out of the two light sources used in this study, the use of the laser light had a higher influence on the growth inhibition of the two bacterial strains involved in halitosis. The materials tested in this study were well tolerated by the human normal gingival fibroblasts, and for P2 samples the cell viability was higher than for C and P1. CPOM investigation showed that the polymer spheres functionalization with silver-graphene powder was effective. The high degree of sphere coating assures an optimal behavior of the material regarding the biological effect evidenced in the research.

The practical aspect of the study could be that PDT using photosensitizer applied on dentures outside the oral cavity followed by their immediate placement into the mouth may be a safer, easier, and more efficient procedure for growth inhibition of different oral bacteria in denture wearers with no risks of pain, mucosal lesions, or other side effects. The results could be helpful especially for very old, disabled, or institutionalized patients. Patients that use orthodontic devices may also benefit from this treatment.

Oral health-promoting programs addressed to denture wearers could improve their oral health. An adequate oral and denture-hygiene routine together with denture overnight removal can reduce bad breath in denture wearers. Association of denture materials with other materials and procedures could be beneficial for obtaining a highly effective antibacterial activity, and for improving oral hygiene. Further studies regarding the exact mechanism of interaction between the light, photosensitizer, PMMA, and oral bacteria and also the influence of the material composition and surface characteristics, exposure time, and light wavelength are necessary. Future research should also explore the in vivo settings.

## 5. Conclusions

Bacterial-intensive growth and halitosis in denture wearers could be more easily and effectively counteracted by using new denture materials in association with non-invasive and clinically safer therapies. The addition of graphene silver nanoparticles to PMMA acrylic resins showed antimicrobial effects on halitosis-responsible bacteria. Extra-oral activation of a photosensitizing agent placed on denture base materials enhanced with graphene silver nanoparticles (1 wt% and 2 wt%) proved to increase the inhibition of bacteria associated with halitosis in acrylic denture wearers, especially when using laser light and higher nanoparticle concentrations (2 wt%). A higher inhibitory effect was found on the *Porphyromonas gingivalis* strain. According to these findings, PDT could be used, in association with materials with antibacterial properties, as a safer and more efficient alternative technique against bad breath and oral infections of denture wearers and of orthodontic device users. It may also be an easier way to control oral and denture hygiene especially for very old, disabled, or institutionalized patients and a possibility to improve their well-being and quality of life. 

## Data Availability

Not applicable.

## Abbreviations

PDT—photodynamic therapy; PET—polyethylene teraphtalate; PMMA—polymethyl methacrylate; H_2_S—hydrogen sulfide; (CH_3_)_2_S—dimethyl sulfide; CH_3_SH—methyl mercaptan; NaOCl—natrium hypochlorite, LED—light emitting diode; LAD—light activated therapy; SEM—scanning electron microscopy; HGF—human gingival fibroblasts, G-Ag graphene silver.
